# AAA+ Ring and Linker Swing Mechanism in the Dynein Motor

**DOI:** 10.1016/j.cell.2008.11.049

**Published:** 2009-02-06

**Authors:** Anthony J. Roberts, Naoki Numata, Matt L. Walker, Yusuke S. Kato, Bara Malkova, Takahide Kon, Reiko Ohkura, Fumio Arisaka, Peter J. Knight, Kazuo Sutoh, Stan A. Burgess

**Affiliations:** 1 Astbury Centre for Structural Molecular Biology and Institute of Molecular and Cellular Biology, University of Leeds, Leeds LS2 9JT, UK; 2 Department of Life Sciences, Graduate School of Arts and Sciences, University of Tokyo, Komaba 3-8-1, Tokyo 153-8902, Japan; 3 MLW Consulting, 11 Race Hill, Launceston, Cornwall PL15 9BB, UK; 4 Graduate School and School of Bioscience and Biotechnology, Tokyo Institute of Technology, 4259 Nagatsuta-cho, Yokohama 226-8501, Japan

**Keywords:** CELLBIO

## Abstract

Dynein ATPases power diverse microtubule-based motilities. Each dynein motor domain comprises a ring-like head containing six AAA+ modules and N- and C-terminal regions, together with a stalk that binds microtubules. How these subdomains are arranged and generate force remains poorly understood. Here, using electron microscopy and image processing of tagged and truncated *Dictyostelium* cytoplasmic dynein constructs, we show that the heart of the motor is a hexameric ring of AAA+ modules, with the stalk emerging opposite the primary ATPase site (AAA1). The C-terminal region is not an integral part of the ring but spans between AAA6 and near the stalk base. The N-terminal region includes a lever-like linker whose N terminus swings by ∼17 nm during the ATPase cycle between AAA2 and the stalk base. Together with evidence of stalk tilting, which may communicate changes in microtubule binding affinity, these findings suggest a model for dynein's structure and mechanism.

## Introduction

Dyneins are large motor proteins that use ATP to power movement toward the minus end of microtubules (MTs) in eukaryotes. Multiple axonemal dynein isoforms drive the beating motions of cilia and flagella (DiBella and King, 2001), whereas cytoplasmic isoforms play important roles in mitosis and trafficking of diverse cargoes within the cell (Hook and Vallee, 2006; Karki and Holzbaur, 1999), including those required for the assembly of cilia and flagella (Pfister et al., 2006). However, despite advances in understanding dynein's motor properties (Gennerich et al., 2007; Kon et al., 2005; Mogami et al., 2007; Reck-Peterson et al., 2006; Shima et al., 2006b), the structure and mechanism of the motor remain poorly understood.

**Figure 1:**
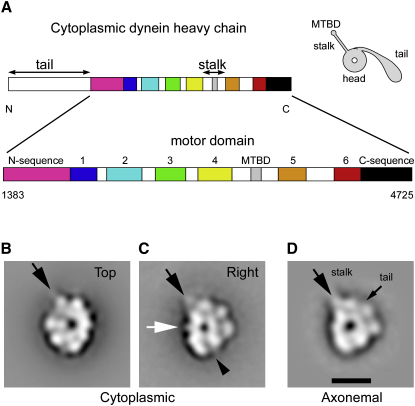
Cytoplasmic Dynein Motor Domain of *D. discoideum* (A) Heavy-chain sequence diagram showing six AAA+ modules (numbered), N and C sequences, and the MT-binding domain (MTBD). On the right, the cartoon shows the stalk-head-tail architecture of dynein (tail formerly known as the stem). (B and C) Negative-stain EM and single-particle image processing of the motor domain reveals two characteristic appearances: top and right views. (D) Right view of axonemal dynein-c from *Chlamydomonas reinhardtii* (modified from Burgess et al., 2003) for comparison. The point of emergence of the coiled-coil stalk (B–D, black arrows) and the tail of dynein-c (D, small arrow) are indicated, as well as a spike (C, white arrow) and stain-filled groove (C, black arrowhead) seen in cytoplasmic dynein (compare with D). The scale bar represents 10 nm.

Cytoplasmic dynein contains two identical heavy chains of ∼500 kDa, each of which possesses motor activity (Nishiura et al., 2004; Reck-Peterson et al., 2006), together with accessory chains associated with dimerization, regulation, and cargo binding (Pfister et al., 2006). The heavy chain (Figure 1A) comprises head, stalk, and tail domains (Samso et al., 1998; Burgess and Knight, 2004). The head contains six concatenated AAA+ modules (Neuwald et al., 1999). AAA1 is the primary site of ATP hydrolysis, whereas AAA2–AAA4 bind nucleotide and appear to be regulatory in function (reviewed in Numata et al., 2008). AAA5 and AAA6 lack nucleotide-binding motifs and are therefore thought to play a structural role. The stalk is located between AAA4 and AAA5 and is an antiparallel coiled-coil structure ending in a microtubule-binding domain (MTBD) (Gee et al., 1997). The N-terminal tail mediates dimerization, and binds accessory chains and cargo. In axonemal dynein-c, a linker domain connects the tail and head and has been interpreted to undergo a nucleotide-dependent powerstroke, switching between two orientations relative to the head (Burgess et al., 2003). However, structural evidence for the linker in cytoplasmic dynein is lacking. Deletion of cytoplasmic dynein's tail leaves a monomeric motor domain of ∼380 kDa (Nishiura et al., 2004; Reck-Peterson et al., 2006; Samso et al., 1998), which is the subject of this study. When the motor domain is artificially dimerized, the resulting molecule can take multiple steps along MTs without detaching and thus retains processive motor function (Reck-Peterson et al., 2006).

None of the subdomains of dynein's head has been solved to atomic resolution. When visualized by electron microscopy (EM), dynein's head has a ring-like appearance characteristic of oligomeric AAA+ proteins (Burgess et al., 2003; Kotani et al., 2007; Mizuno et al., 2007; Samso et al., 1998). However, dynein is unusual in having its six AAA+ modules covalently linked. The AAA+ modules are thought to be arranged sequentially around the head (King, 2000; Mocz and Gibbons, 2001; Serohijos et al., 2006), and support for this arrangement for AAA1–AAA4 has been obtained (Takahashi et al., 2004). However, current structural data do not define the positions of AAA+ modules within the head, so their organization relative to one another and to other subdomains remains to be elucidated.

C-terminal to AAA6 is a region (referred to here as the C sequence; Figure 1A) of unknown fold but essential for motor function (Gee et al., 1997). Fungal dynein isoforms have shorter C sequences (of ∼15 kDa) that correspond to the first part of the longer C sequences (∼46 kDa) of other dyneins (Mocz and Gibbons, 2001). EM studies suggesting seven lobes of density around the head (Burgess and Knight, 2004; Koonce and Samso, 2004) have led to a model in which the AAA+ modules are arranged sequentially, with the C sequence forming a seventh domain between AAA1 and AAA6 (Hook and Vallee, 2006; King, 2000; Mizuno et al., 2007; Oiwa and Sakakibara, 2005; Serohijos et al., 2006). However, this heptameric model is untested.

N-terminal to AAA1 is an ∼60 kDa region (referred to here as the N sequence; Figure 1A) also essential for motor activity (Gee et al., 1997; Koonce and Samso, 1996). ATP-dependent movement of the N terminus of the motor domain relative to the head has been suggested by fluorescence resonance energy transfer (FRET) studies using green fluorescent protein (GFP) and blue fluorescent protein (BFP) -tagged motor domains (Imamula et al., 2007; Kon et al., 2005; Mogami et al., 2007). These FRET studies define two major conformations of the motor, referred to here as primed and unprimed. Binding of ATP is associated with the priming stroke. Release of hydrolysis products triggers the powerstroke when the motor is bound to MT, or an unpriming stroke when the motor is free in solution. In vitro motility studies suggest that the N sequence has a lever-like function, acting as the main transmitter of mechanical motion during these transitions (Reck-Peterson et al., 2006; Shima et al., 2006b). This is consistent with a linker swing model in which the N sequence corresponds to the linker and undergoes a powerstroke (Numata et al., 2008). However, a recent EM study was interpreted as showing the motor N terminus of cytoplasmic dynein at random positions around the head, arguing that the N sequence in this species is instead a highly flexible linkage to the cargo (Meng et al., 2006). Therefore, structural evidence for N sequence function is currently controversial.

Here we map by negative-stain EM the positions of key sites within the cytoplasmic dynein motor domain of *Dictyostelium discoideum*. We use GFP-dynein fusion proteins, including those used in the earlier FRET study (Kon et al., 2005) to map the positions of the motor N terminus in primed and unprimed conformations and identify the positions of six other sites within the unprimed head. The tags are β barrel proteins (Yang et al., 1996), which we locate by EM. We also report the structure of truncation constructs in which the N and C sequences are removed. From these data, we present a model for the subdomain organization and mechanism of dynein.

## Results

The motor domain of cytoplasmic dynein in the unprimed conformation adopts two orientations on the EM grid under our negative staining conditions, giving two distinct ring-like views (Figures 1B and 1C). The most common view (Figure 1B) is similar to previous images of an identical motor construct (Samso and Koonce, 2004). We refer to this here as the “top” view because it corresponds to the view looking directly along the channel axis in their 3D reconstruction. With the image oriented so that the stalk emerges at the 11 o'clock position, well-defined stain-excluding lobes are visible around the left side of the head, while density on the other side is stronger but less distinct. The second view (Figure 1C) resembles the “right” view of axonemal dynein-c (Figure 1D) (Burgess et al., 2003). The right view similarly oriented (Figure 1C) shows pronounced stain-excluding lobes around the upper and right side of the head and less differentiated density on the left side. A spike and a groove (Figure 1C, arrowheads) are pronounced in the cytoplasmic dynein. However, the main conclusion from these first images of right views of cytoplasmic dynein is that the head structures of axonemal and cytoplasmic isoforms are strikingly similar.

### Location and Structure of the Stalk in the Unprimed Motor

**Figure 2:**
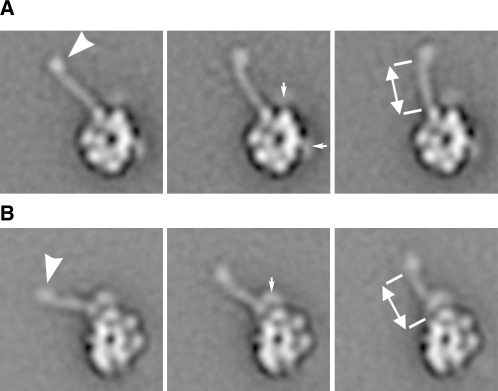
Stalk Structure in the Unprimed Motor Class averages of (A) top and (B) right views showing the stalk at a range of angles (compare left, middle, and right panels) with the MTBD at the distal end (arrowhead), which is often curved to the right. Diffuse stain-excluding areas (small arrows) are the GFP and BFP of this construct (GN-motor-B2). Coiled-coil lengths of 10.3 ± 0.6 nm (top view, mean ± SD, n = 10 classes) and 10.5 ± 0.7 nm (right view, n = 10 classes) were measured as indicated by double-headed arrows. For further details, see legend to Movie S3.

Image classification reveals that the MT-binding stalk emerges at a range of angles from a fixed position in the head (Figure 2; see Movie S3 available online). In top views it emerges from a prominent lobe of density, whereas in right views it emerges between two adjacent lobes (see also Figure 1C). The stalk is about 2 nm wide, consistent with the prediction of a coiled-coil structure. The visible coiled coil is 10.4 nm long. With the ∼4 nm distal MTBD this is very similar to the length of dynein-c's stalk (Burgess et al., 2003). The coiled coil of dynein-c in the unprimed conformation has a bend about two-thirds along its length (Burgess et al., 2003), which may correspond to a proline residue within the outward α helix (Yagi et al., 2005). By contrast, the stalk of cytoplasmic dynein lacks this proline and is straight, except for an occasional kink (to the right) at its distal end immediately adjacent to the MTBD (Figure 2).

### Dynein's Six AAA+ Modules Alone Form a Ring

**Figure 3:**
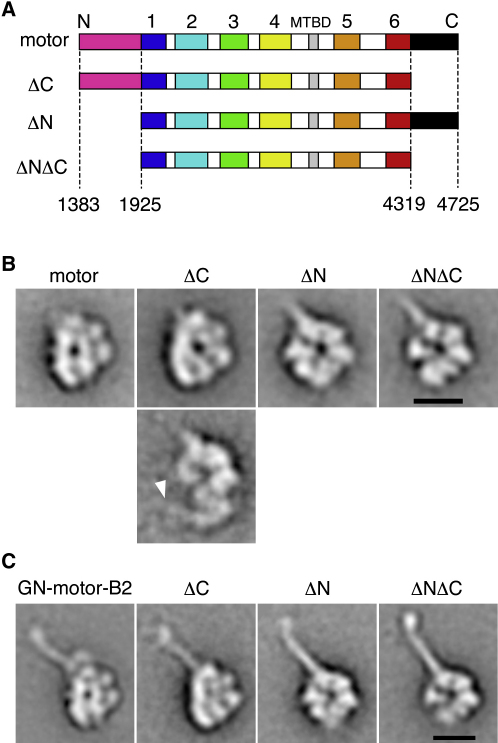
Structural Impacts of Truncation on the Dynein Motor (A) Sequence diagrams of the motor domain and truncation constructs lacking the C sequence (ΔC), the N sequence (ΔN), and both (ΔNΔC). (B) Class averages showing right view of the motor domain alongside ΔC, ΔN, and ΔNΔC constructs. ΔC molecules resembling the motor domain in right view (upper panel) and with the linker undocked (arrowhead, lower panel) are shown. (C) A stalk emerges from each head at the same position (∼11 o'clock). The scale bars represent 10 nm.

To investigate the structure of the head domain, in particular the contribution made by the AAA+ region, we engineered truncation constructs lacking the C sequence (ΔC), the N sequence (ΔN), and both these flanking sequences (ΔNΔC; see Figure 3A). Functional assays showed that the C sequence is not required for basal ATPase but is required for MT-binding and motile activities, whereas the N sequence is required for all these activities (Table S1). Analytical ultracentrifugation showed that all three truncation constructs retain a low frictional coefficient similar to the intact motor domain, indicative of a compact fold (Table S1).

EM and image processing reveal that the ΔC construct, which lacks all 406 residues C-terminal to AAA6, is strikingly similar to the motor domain in the right view (Figure 3B). The head has the same overall asymmetric ring shape and the stalk is present in the same place (Figure 3C). The main difference is the appearance of reduced density to the right of the central stain pool and a loss of the spike on the left margin of the head (Figures 3B and 3C). The ΔC construct does not show a gap in the head corresponding to a missing peripheral domain that would be expected from heptameric models.

A subset of ΔC molecules (∼8%) has a different appearance. An extended lobe of material protrudes from the head opposite the stalk (arrowhead, Figure 3B) and is variable in position. This structure has appropriate dimensions to be the linker previously identified in axonemal dynein-c (Burgess et al., 2003). Such images suggest that deletion of the C sequence can destabilize linker-head interactions to favor linker undocking.

ΔN molecules, which lack the 542 residues N-terminal to AAA1, show a striking new “ring” appearance, rather than top or right views (Figure 3B). This new appearance is more symmetrical, with wedge-shaped densities defined by radial lines of stain. The stalk is intact (Figure 3C) and emerges from one of the wedge-shaped densities.

ΔNΔC molecules, which lack both N and C sequences, also show a ring appearance. The ΔNΔC ring is surprisingly similar to the ΔN ring (Figure 3B), despite the loss of the C sequence, equivalent in length to two AAA+ modules. The main difference between ΔNΔC and ΔN is weaker density at the ∼8 o'clock position (as orientated in Figure 3B), and increased variability in this region, as indicated by less sharply defined density here (Figure 3C). Overall, the ring appears complete and the stalk is intact (Figure 3C).

These truncation constructs reveal several new aspects of the organization within the dynein head. The C sequence is not an integral part of the ring as proposed in heptameric models. Instead, the C sequence may stabilize closure of the ring because its removal causes structural variability opposite the stalk. Removal of the N sequence reveals a more symmetrical ring, recalling images of other ring-shaped AAA+ proteins (Mocz and Gibbons, 2001). Together, these results show that dynein's six AAA+ modules alone form a ring structure.

### Mapping Sites within the Motor Domain Using GFP-Based Tags

**Figure 4:**
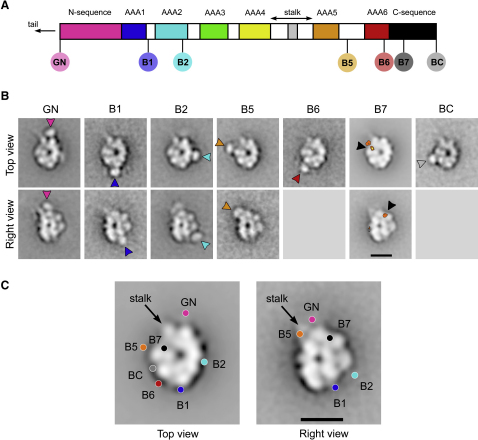
Mapping the Locations of the GFP-Based Tags in Dynein Fusion Proteins in the Unprimed Conformation (A) Motor domain sequence (residues 1383–4725) showing sites of insertion of GFP and BFP in the constructs examined (GN, inserted upstream of V1383; B1, after A2172; B2, after S2471; B5, after K3928; B6, after E4261; B7, after S4450; BC, after I4725). (B) Class averages of top and right views showing each tag (arrowheads) near its mean position. The internal position of the B7 tag is shown by difference mapping (see Figure S2 for details) which, because of superposition with the head, means that the BFP could not be shown by image classification. Difference maps are contoured at 5σ above the mean then at intervals of 2σ, superposed on global averages faded for clarity. No right views were obtained for B6- and BC-tagged constructs. (C) Summary of GFP-based mapping. Mean positions of GN, B1, B2, B5, B6, and BC tags and peak of difference maps for B7 tag are shown (colored circles). The scale bars represent 10 nm.

To determine how the heavy-chain sequence maps onto the morphology of the motor domain, we used EM to examine fusion proteins in which GFP and BFP were inserted at seven different locations (Figure 4A; Figure S1). These tagged constructs show robust MT-sliding activity (Kon et al., 2005), including those newly engineered in this study (data not shown). Images of tagged motors (Figure 4B) appear substantially similar to the untagged motor (Figures 1B and 1C), indicating that insertion of the tags does not perturb the overall fold of the heavy chain.

We used two methods to establish the location of the tags: difference mapping (Figure S2) and a novel image classification procedure to scan systematically positions around the perimeter of the head (Movies S1 and S2). The results from these two methods are consistent. We then applied image classification to the regions identified by the first two methods, to show the tags in more detail (Figure 4B). The tags appear as globular densities consistent with the β barrel structures of GFP and BFP. To locate the tags accurately and without bias, we used an automatic detection procedure (see legend, Figure S2) and calculated their mean positions (Figure 4C), which we describe in detail below.

### The N Terminus of the Unprimed Motor Lies near the Stalk Base

The GFP tag fused to the motor N terminus is close to the periphery of the head near the base of the stalk (Figure 4B; Figure S2). This is observed in both top and right views. Scanning classification confirms that N-terminal GFP is absent from other positions around the perimeter of the head (Movies S1 and S2). This finding is contrary to an earlier suggestion (Meng et al., 2006) that the motor N terminus is randomly orientated around the head and lies at a high radius (see legend to Movie S2 for discussion). We conclude that in the unprimed conformation the N terminus lies near the base of the stalk. This location is close to the linker-tail junction in axonemal dynein-c (Figure 1D), suggesting that a similar linker exists in cytoplasmic dynein.

### AAA1 Is Opposite the Stalk and the N Sequence Spans the Head

The B1 tag, inserted 20 amino acids downstream of the main catalytic AAA+ module (AAA1), has a peripheral location opposite the stalk in both top and right views (Figures 4A and 4B). Because GN and B1 tags lie on opposite sides of the head, the polypeptide chain between them must span the head. Within this sequence, ∼240 amino acids are predicted to form AAA1 and the downstream sequence to B1 (Figure 4A), leaving the remaining ∼550 amino acids upstream of AAA1 to span ∼14 nm across the head (Figure 4C). This fits the model in which the N sequence includes the linker domain (Numata et al., 2008), the mechanical lever originally proposed by Burgess et al. (2003).

### AAA2, AAA5, and AAA6 Fit a Counterclockwise Arrangement of AAA+ Modules

Having established that AAA1 lies opposite the stalk and N terminus, we next determined the direction of AAA+ modules around the head. The B2 tag, inserted within AAA2, is positioned counterclockwise from the B1 site in top and right views (Figure 4B). This indicates that both views show the same face of the AAA+ ring. Confirming this, the B5 tag inserted 68 amino acids downstream of AAA5 lies counterclockwise of the stalk in both views. The B6 tag, inserted within AAA6, lies counterclockwise from the B5 site in top views (Figure 4B). Thus, in the views shown, AAA1, AAA2, AAA5, and AAA6 are arranged counterclockwise around the ring (Figure 4C). The close proximity between AAA1 and AAA6 fits our finding that the core of the motor is a hexameric ring of AAA+ modules.

### The C Sequence Spans between AAA6 and near the Stalk Base

The finding that the C sequence does not close the ring raises the question: where is it located within the head? To investigate this we located the B7 tag, inserted about one-third through the C sequence (corresponding approximately to the naturally truncated C terminus of fungal dyneins). Difference mapping shows that the B7 tag has an internal position within the head in top and right views (Figure 4B) in contrast to the other tags. In both views, B7 is located within ∼6 nm of the base of the stalk. To map where the C sequence terminates, we imaged a new construct with BFP fused at the C terminus of the motor (BC). The BC tag lies on the head periphery between the B5 and B6 tags (Figure 4B). Thus, the C sequence spans from AAA6 toward AAA5 and the base of the stalk in its first one-third and then returns toward AAA6 (Figure 4C).

### Movement of the Linker during the Priming Stroke

**Figure 5:**
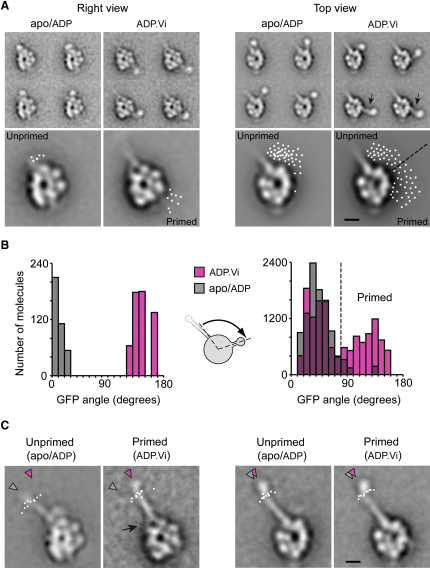
Structural Changes between Unprimed and Primed Conformations (A) Motor tagged with GFP at the N terminus and BFP at the B1 site in apo/ADP and ADP.Vi states. Small panels: representative class averages showing N-terminal GFP and distal linker (arrows). Larger panels: GFP positions detected automatically (white spots). In ADP.Vi-motors in top view, GFP positions are segregated into primed and unprimed positions (dashed line; see B). (B) Histograms showing angular position of N-terminal GFP measured clockwise relative to an axis passing through the head center and the base of the stalk (cartoon). Mean GFP angle in right view is 10° ± 11° (n = 375) in apo/ADP compared to 145° ± 11° (mean ± SD, n = 557 molecules) in ADP.Vi. Gaussian fits to the top-view data in ADP.Vi give two peaks for GFP angles of 41° ± 18° (mean ± SD) and 124° ± 25°, which intersect at 80° (dashed line). The low-angle peak coincides with that of unprimed motors (also 41° ± 18°). The numbers of molecules in top views are 9,964 (apo/ADP) and 13,527 (ADP.Vi). (C) Average stalk angles show a clockwise tilt between unprimed and primed motors (gray and magenta arrowheads, respectively). Stalk angles: right view: unprimed −16° ± 9° (mean ± SD, n = 3858 molecules), primed 0° ± 8° (n = 526); top view: unprimed −6° ± 6° (n = 1604), primed −4° ± 6° (n = 1667). White spots show positions of distal coiled coil in each of the ten classes used to obtain these values (see also Movie S3). Bifurcation of the stalk is indicated (arrow). Right-view unprimed motor is tagged with BFP at the B2 site rather than at the B1 site. The scale bars represent 5 nm.

To investigate dynein's motile mechanism, we located the position of the GFP tag attached to the linker N terminus in the unprimed and primed conformations. To generate the primed conformation, we treated the motor with ATP and vanadate to trap the ADP.Vi complex (Kon et al., 2005). Most strikingly, in ADP.Vi motors, GFP is shifted toward AAA2 (Figure 5A). This is seen in both right view (4% of motors) and top view (96%), although the distribution of GFP positions differs in these two views. In right view, all ADP.Vi motors show GFP close to AAA2 (Figure 5B). In top view, the distribution is bimodal: 44% show GFP near AAA2, while 56% show GFP near the stalk base, coinciding with its unprimed location (Figure 5B). This leads us to speculate that in the ADP.Vi motor (1) the linker exists in a poised equilibrium between primed and unprimed conformations and (2) the equilibrium position is altered by the orientation of the molecule on the EM grid. This might be analogous to the situation in other motors, where crystallization conditions are thought to shift conformational equilibria of myosin's converter domain and kinesin's neck linker (Vale and Milligan, 2000). Our 3D analysis (below) shows that in the top view, but not the right view, the linker swing has a large component in the z direction, suggesting that the top view is the more likely of the two to have been influenced by the EM grid. The distal portion of dynein's linker is occasionally revealed in top views of ADP.Vi motors, as a rod ∼2 nm thick (Figure 5A, arrows) connecting the N-terminal GFP to the head (close to the B2 site). Together, these observations suggest that during the priming stroke, the motor N terminus moves from near the stalk base and AAA4 toward AAA2 by a swing of the linker.

**Figure 6:**
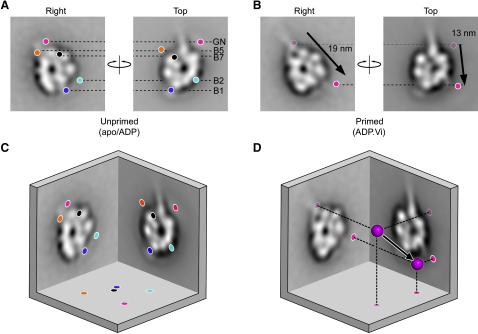
Magnitude and Direction of the Linker Swing In this figure, right and top views of the dynein head are related by a rotation about the y axis (see Figure S3 for details): each view has been rotated in the plane of the page relative to previous figures so that tag positions move only in the x direction (dashed lines). (A) Tag positions in the unprimed motor are indicated by colored spots (as in Figure 4C). (B) For the primed motor, the mean position of the N-terminal GFP tag (brighter magenta spots; derived for top view as explained in the main text) also moves only in the x direction. Magnitudes of the displacements from the unprimed positions (faded magenta spots) are indicated (arrows). (C and D) Top and right views are related by a rotation of between 50° and 116° (see Figure S4 for details): illustrated here is a rotation of 90° (see also Movie S4). Tag positions in the unprimed conformation (C) and N-terminal GFP in the unprimed and primed conformations (D). In each case, the bottom surface of the cube shows their projected positions perpendicular to top and right views. The black arrow shows movement of N-terminal GFP during the priming stroke, which has a major component perpendicular to the plane of the top view and parallel to the plane of the right view. The 3D displacement of N-terminal GFP lies between 18.8 and 21.1 nm (depending on the rotation angle between right and top views).

The mean displacement of GFP during the priming stroke is 19 nm in right views (Figure 6B). Measurement in top views is complicated by the broader distribution of GFP in ADP.Vi motors. Based on Gaussian fits to the bimodal distribution of GFP angles around the head (Figure 5B), we segregated the ADP.Vi motors into two subpopulations (Figure 5, legend). We define the motors in the subpopulation nearer AAA2 as the primed conformation. Using the mean GFP position of this subpopulation, the displacement of GFP during the priming stroke is 13 nm in top views (Figure 6B). In both views, the direction of the linker swing is almost parallel to the long axis of the stalk.

Right views of ADP.Vi motors show a prominent accumulation of stain at the base of the stalk (Figure 5C, arrow), not seen in apo/ADP motors. This change at the stalk base likely occurs because of movement of the linker N terminus. This accumulation of stain suggests that the stalk coiled coil bifurcates at the junction with the head (Figure 5C, arrow), as reported for dynein-c (Burgess et al., 2003).

### Tilting of the Stalk between Weak and Strong MT-Binding States

The ADP.Vi motor binds to MTs with weaker affinity than the apo/ADP motor (Imamula et al., 2007). Between these states, we find that the angle of the stalk changes relative to the head (Figure 5C). In both states the stalk angle is variable (Movie S3), with similar standard deviations (Figure 5C, legend). The distributions overlap but there is a shift in their mean angles. From apo/ADP to ADP.Vi, the stalk tilts clockwise: in right view by 16° and in top view by 2° (Figure 5C), the former displacing the center of the MTBD by 5 nm. Thus, relative to the head, the stalk undergoes a small nucleotide-dependent tilt.

### Three-Dimensional Movement of the Linker N Terminus during the Priming Stroke

To determine the positions of the various tags in 3D, and the 3D movement of the linker N terminus, we calculated the angular relationship between top and right views (Figures S3 and S4). Superficially, top and right views look like reflections of one another in a vertical mirror, suggesting they may be related by a rotation of ∼180°, but the similar emergence points of the stalk and the positions of the tags, most notably B2 and B5, rule this out (Figure 4C). To obtain the axis of rotation between top and right views, we used the positions of tags in the unprimed motor (Figure S3). The axis of rotation obtained (Figure 6A) is also compatible with the segregation in top views of primed and unprimed linker conformations as defined above (Figure 6B). We then used geometric constraints to establish that top and right views are related by a rotation of between 50° and 116° about this axis (see Figure S4). This is consistent with our earlier interpretations of left, side, and right views of dynein-c (Burgess et al., 2004b; see Figure S5). Based on this angular range, we calculated the 3D positions of the five tags (Figures 6C and S4D; see also Movie S4) including the 3D position of GFP in the primed conformation (Figure 6D; Movie S4). This analysis shows that during the priming stroke the majority of GFP movement occurs in the plane of the right view, with a large component perpendicular to the plane of the top view (Figure 6D). The 3D displacement of GFP is ∼19–21 nm (Movie S4). Accounting for the size of GFP, we infer that the distance moved by the linker N terminus is ∼16–18 nm.

## Discussion

### A Model for the Dynein Motor

**Figure 7:**
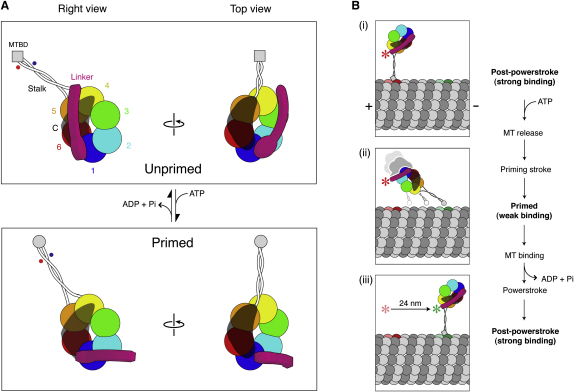
Model for the Structure and Priming Stroke of Dynein (A) Six AAA+ modules (numbered) form a hexameric ring. The C sequence (translucent black) is represented speculatively as an elongated structure interacting with (one or another face of) AAA6, AAA5, and AAA4. The N sequence (magenta) contains the linker which runs from AAA1 across the head to AAA4 (yellow) in the unprimed conformation and switches to a position close to AAA2 in the primed conformation, thereby moving the N terminus of the motor by ∼17 nm (in right view in a plane parallel to the page). The N sequence may also contain nonlinker structures, such as near the junction with AAA1 (magenta ellipse). Tilting of the stalk, shown here to occur entirely in the plane of the page in right view (see Figure S3), displaces the MTBD by ∼5 nm and could shift the registration of the two α helices of the coiled coil (indicated by red and blue spots). Stalk tilting perpendicular to the page is not seen in top-view data (Figure 5C), probably because the stalk flattens down onto the EM grid in this orientation. (B) Model to illustrate how the linker swing and stalk tilt could produce one of dynein's larger displacements along an MT (see Discussion for further details). The attachment geometry proposed here (i and iii) is compatible with Mizuno et al. (2007) and uses the right view, which ensures that movement of the linker N terminus occurs in a plane parallel to the MT axis. With the linker N terminus initially restrained (red asterisk), perhaps by attachment to the second head as observed in dimeric dynein-f (Kotani et al., 2007), ATP binding (ii) causes MT detachment and the priming stroke which displaces the MTBD along the MT, here by 24 nm. Subsequent reattachment and powerstroke (iii) displaces the linker N terminus by 24 nm (green asterisk). α-β tubulin dimers are shown (to scale) as pairs of dark and light gray spheres with red and green dimers showing initial and final binding sites, respectively.

Our protein engineering and EM data suggest a new model for cytoplasmic dynein (Figure 7A). In this model the core of the motor is a hexameric AAA+ ring, with the N sequence and C sequence forming subdomains that interact with the ring. Our new model is a revision of heptameric models in which the C sequence closes the ring. It indicates that the route of energy transduction derived from an atomic heptameric model of dynein (Serohijos et al., 2006) is without empirical foundation. The similarity we find between *D. discoideum* cytoplasmic dynein and *Chlamydomonas reinhardtii* axonemal dynein-c (Figures 1C and 1D) and dynein-f (Kotani et al., 2007) argues that, despite over 800 million years of separate evolution (Cavalier-Smith, 2006), the overall arrangement of subdomains within the dynein motor has been conserved. Therefore, this is a model for all dynein isoforms. The hexameric AAA+ ring of dynein suggests that its mechanism may have parallels with other hexameric AAA+ mechanoenzymes. However, unlike these other AAA+ proteins, activity within dynein's AAA+ ring requires three other components: the stalk, the C sequence, and the N sequence.

### Allosteric Communication between Sites of ATP Hydrolysis and MT Binding

We have shown that AAA1, the main ATPase site linked to force generation (Kon et al., 2004) and control of MT binding (Imamula et al., 2007), is located in the ring opposite the stalk (Figure 7A). This establishes that, compared to myosin and kinesin, dynein's motor has an exceptionally long-range communication pathway. Two-way coupling between the hydrolysis cycle in AAA1 and changes in MT-binding affinity at the tip of the stalk requires transmission both across the ring and along the stalk, a distance of ∼25 nm. In axonemal dynein-c, a nucleotide-dependent structural change within the stalk was reported, involving an increase in curvature and a decrease in stiffness in ADP.Vi (Burgess et al., 2003). Interestingly, the changes in stalk structure seen in cytoplasmic dynein are different. Here we find that the stalk tilts relative to the head between weak and strong binding states. Stalk tilting could occur as a rigid-body motion, that is, without any change in the relative positions of the two α helices of the coiled coil. Alternatively, the two α helices could undergo shear during tilting. Consistent with the latter possibility, evidence suggests that changes in registry between the α helices alter the MT-binding affinity of the stalk (Gibbons et al., 2005). In stalks with different amino acid sequences, such as those of axonemal dynein-c and cytoplasmic dynein, this same mechanism could underlie the different structural changes observed.

### Location and Role of the C Sequence

Our data show that the C sequence is not an integral part of dynein's ring structure. Consistent with this, the C sequence is not an absolutely conserved component of the motor. Fungal isoforms naturally lacking the C-terminal two-thirds of the C sequence still exhibit processive MT stepping (Reck-Peterson et al., 2006), suggesting that the missing segment is not essential for motility. The conserved N-terminal one-third of the C sequence spans a region of the ring from AAA6 toward the stalk base. This implies that in all dyneins, the C sequence may overlap and interact with AAA6, AAA5, and possibly also AAA4 (Figure 7A). The C sequence in *Dictyostelium* (Table S1) and in rat cytoplasmic dynein (Gee et al., 1997) is required for dynein's ability to bind MTs cyclically. By extending to near the stalk base, the C sequence may be positioned to exert allosteric control through the stalk or through the AAA+ ring.

### Structure and Role of the Linker

The N sequence of the motor includes the linker: a lever-like structure first described in an axonemal dynein (Burgess et al., 2003). In the unprimed conformation the linker runs across the head from AAA1, terminating near the stalk base, AAA4, and AAA5. During the priming stroke the linker swings to a position close to AAA2 (Figure 7A). These findings explain previous biochemical and biophysical data. First, loss of motor function following truncation within the N sequence (Gee et al., 1997; Reck-Peterson et al., 2006) is consistent with a disruption of interactions between the linker and the head. Even small N-terminal truncations into the linker sequence disrupt motor activity (ΔN′ construct, Table S1; Reck-Peterson et al., 2006). This distinguishes dynein's mechanical lever from that of myosin, which can be truncated without impairing ATPase (Kurzawa et al., 1997). Second, in vitro motility data showing progressively reduced translocation velocities in constructs tethered to the substrate at various points within the N sequence (Shima et al., 2006b) can now be interpreted in terms of its elongated structure. Third, the proximities between the N-terminal GFP tag and BFP tags within the head (Table S2) show good agreement with estimates based on FRET efficiencies from the same constructs (Kon et al., 2005). Our structural data demonstrate that the nucleotide-dependent FRET changes correspond to an ∼17 nm swing of the motor N terminus and that this occurs predominantly in a plane parallel to the right-view plane.

In our model, priming and unpriming strokes swing the linker between AAA4 and AAA2, and across AAA3 (Figure 7A). Our ADP.Vi data suggest that in ADP.Pi-dynein, the linker may be in equilibrium between primed and unprimed positions (Figure 5A) and therefore sensitive to external force. It is noteworthy that AAA2, AAA3, and AAA4 each bind nucleotide in a manner that regulates dynein function (Kon et al., 2004). Taken together, these findings suggest the possibility that external load, linker position, and regulatory nucleotide binding may be coupled.

### Dynein's Priming Stroke and Implications for Stepping

How might this new structural information help us understand how dynein steps along MTs? The attachment geometry of two-headed cytoplasmic dynein bound to MTs is not yet known. However, the attachment geometry of single-headed cytoplasmic dynein in the strongly bound (i.e., postpowerstroke) state has been observed (Mizuno et al., 2007). In vitro motility studies have shown that two-headed dynein molecules step along MTs with center-of-mass displacements predominantly of 8 nm (the spacing between tubulin dimers) interspersed with larger steps up to 32 nm, as well as backward steps and off-axis steps (Gennerich et al., 2007; Reck-Peterson et al., 2006). This distinguishes dynein from the other MT motor kinesin, which takes steps of 8 nm with high regularity. During dynein stepping, center-of-mass displacements report the movement of the fused N termini of artificially dimerized motor domains, which are therefore expected to correspond quite closely to movement of the linker N terminus. Our new structural data showing movement of the linker N terminus in 3D suggest an MT-docking geometry for our dynein model (see Figure 7B and legend for further details). According to this model the right view lies in the plane that includes the MT axis, and the swing of the linker N terminus and the tilting of the stalk occur almost entirely in this plane.

Our MT-bound model can account for dynein's distinguishing ability to take larger steps (Figure 7B). If the N terminus of the linker is held in place during the priming stroke, rotation of the head and stalk against the linker can displace the MTBD by 24 nm along the MT (Figure 7Bii). Compliance in the motor, for example in the stalk or between stalk and head (Movie S3), could allow even greater reach along the MT and also off-axis, around the MT. On the other hand, a submaximal priming stroke would restrict the search range along the MT, leading to smaller steps (e.g., 8 nm), typical of the dimer (Toba et al., 2006; Reck-Peterson et al., 2006). Our finding that movement of the linker N terminus occurs roughly parallel to the long axis of the stalk suggests that the priming stroke causes the MTBD to skate along the MT surface (Figure 7Bii). Gennerich et al. (2007) have proposed that the affinity of the MTBD for MT is sensitive to the angle between the stalk and the MT. We favor this model, because the low stalk-MT angle demanded for larger steps (Figure 7Bii) would disfavor MT reattachment, thereby biasing the motor toward smaller steps. During the subsequent powerstroke a swing of the linker toward AAA4 pulls cytoplasmic dynein forward (Figure 7Biii). In this model, dynein acts like a winch (Burgess and Knight, 2004) with the linker acting as the crank. This model is compatible with previous functional studies (Gennerich et al., 2007; Imamula et al., 2007; Mogami et al., 2007; Reck-Peterson et al., 2006; Shima et al., 2006b), but further such studies are necessary to test specific features of it.

In flagellar outer-arm dyneins containing either two or three heads, the rings appear stacked upon one another in situ (Ishikawa et al., 2007; Lupetti et al., 2005; Nicastro et al., 2006; Oda et al., 2007), implying that in these systems the two or three linkers could have different environments, with some sandwiched between two adjacent rings. This poses the intriguing possibility that during their priming and power strokes the linker swing might be restrained or regulated. Whether ring-ring stacking occurs in cytoplasmic dyneins remains unknown. Higher-resolution 3D structures of the motor domain and of two-headed dynein bound to MTs will be necessary to answer some of these outstanding questions. The subdomain mapping and conformational changes reported here will aid interpretation of such new data.

## Experimental Procedures

### Protein Engineering, Expression, and Purification

*D. discoideum* cytoplasmic dynein constructs (summarized in Figure S1) were prepared as described (Kon et al., 2004, 2005; Nishiura et al., 2004). The 380 kDa motor domain (V1383–I4725) was fused with N-terminal His6, FLAG, and biotinylation tags (Shima et al., 2006a). To create an N-terminal GFP-tagged motor (GN-motor), GFP and a spacer sequence (GGGK) were inserted in place of the biotinylation tag. To create dual-tagged constructs (GN-motor-BFP), BFP was additionally inserted within the motor via flanking spacer residues (N-terminal TGGG and C-terminal GGGTG) as described (Kon et al., 2005). The new dual-tagged constructs used in this study (GN-motor-B6 and GN-motor-BC) were engineered with BFP within AAA6 (after E4261) or at the C terminus (I4725), respectively. A new single-tagged construct (motor-B5) was engineered with a PreScission protease cleavage sequence (TGGGSLEVLFQGPGG) followed by BFP downstream of AAA5 (after K3928).

Truncation mutants ΔC (V1383–I4319), ΔN′ (G1459–I4725), ΔN (A1925–I4725), and ΔNΔC (A1925–I4319) were engineered with N-terminal His6, FLAG, and biotinylation tags. For FRET assays on ΔC and ΔN′, GFP at the N terminus and BFP at site B2 within AAA2 were engineered as described (Kon et al., 2005).

Dynein constructs were expressed in *D. discoideum* and purified by Ni-NTA affinity followed by MT or FLAG affinity (Kon et al., 2005). Elution was in PMEG buffer (30–100 mM K-PIPES, 4 mM MgCl_2_, 5 mM EGTA, 0.1 mM EDTA, 8.3% glycerol [pH 7.0]) containing 1 mM DTT, 10 μg/ml chymostatin, 10 μg/ml pepstatin, 50 μg/ml leupeptin, 0.5 mM PMSF, and 0.1–10 mM ATP, with 200 μg/ml FLAG peptide where required.

### Biochemical Assays

Basal and MT-activated ATPase, MT-binding, MT-sliding, and FRET of dynein constructs were measured as described (Imamula et al., 2007; Kon et al., 2004, 2005; Shima et al., 2006a). Analytical ultracentrifugation was performed as described (Zhao et al., 2003), and frictional coefficients were determined using f ∝ M/S for each species (f, frictional coefficient; M, molecular weight; S, sedimentation coefficient).

### Sample Preparation and Electron Microscopy

Dynein constructs were prepared for negative-stain EM as described (Burgess et al., 2004a). Constructs were diluted to ∼40 nM with either buffer 1 (20 mM K-MOPS, 20 mM KCl, 5 mM MgCl_2_, 1 mM EGTA [pH 7.4]), buffer 2 (10 mM K-PIPES, 50 mM K-acetate, 4 mM MgSO_4_, 1 mM EGTA [pH 7.0]), or buffer 3 (30 mM K-MOPS, 15 mM KCl, 2 mM MgSO_4_, 0.2 mM EGTA, 16% v/v methanol [pH 7.4]). No differences in dynein structure were observed between these different buffer conditions.

Samples were negatively stained with 1% uranyl acetate on carbon-coated grids that had been freshly UV treated. For the unprimed conformation, samples were either pretreated with apyrase (10 U/ml for 30 min or 24 hr on ice) to remove ADP and ATP, or used directly after dilution (all ATP in the PMEG buffer had been hydrolyzed to ADP, as confirmed by HPLC [data not shown], giving a final ADP concentration of 4–666 μM). To trap constructs in the primed conformation, samples were treated with 200 μM ATP and 200 μM sodium orthovanadate (residual ADP concentration was 4 μM). FRET assays with GN-motor-BFP constructs were used to confirm that these treatments generated the primed and unprimed conformations, as described (Kon et al., 2005).

Micrographs were taken at 40,000× nominal magnification and calibrated using the paramyosin spacing of 14.4 nm (Elliott et al., 1976) on a JEOL 1200 EX operating at 80 kV with an LaB_6_ electron source. Micrographs were digitized on an Imacon Flextight 848 scanner (Hasselblad A/S, Copenhagen, Denmark), giving a final object sampling of 0.504 nm/pixel.

### Image Processing

Digitized images were imported into either SPIDER (Frank, 2006) or BOXER (Ludtke et al., 1999) for manual or automatic identification of particles, respectively. Particles were cut out from micrographs and aligned in SPIDER using reference-free methods and classified using IMAGIC (Image Science Software GmbH, Berlin, Germany) or SPIDER as described (Burgess et al., 2004a). Details of image classification can be found in figure legends in Supplemental Data. Classes showing well-stained top, right, or ring views were selected manually and processed separately in subsequent steps. The number of particles analyzed is as follows: 11,504 (control motor); 10,863 (GN-motor); 12,987 (GN-motor-B1); 34,625 (GN-motor-B2); 34,875 (motor-B5); 10,810 (GN-motor-B6); 19,284 (GN-motor-B7); 7,628 (GN-motor-BC); 13,027 (ΔC); 4,407 (ΔN); 9,648 (ΔNΔC); 26,298 (GN-motor-B1 primed); and 19,750 (GN-motor-B1 unprimed); 215,706 (total).

### Orientation of Top and Right Views

The axis of rotation relating top and right views was obtained by sinogram analysis (van Heel et al., 2000) in IMAGIC using the mean positions of GFP and BFP (GN, B1, B2, and B5) and the maxima of the difference maps for B7 in the unprimed motor (for details, see Figure S3). The magnitude of rotation about this axis was estimated by constraining simultaneously the radius of all tags to lie within 12.0 nm of the rotation axis (for details, see Figure S4).

## Note Added in Proof

Since acceptance of this manuscript, a paper has been published describing the crystal structure of the MTBD and distal coiled coil of a cytoplasmic dynein (Carter, A.P., Garbarino, J.E., Wilson-Kubalek, E.M., Shipley, W.E., Cho, C., Milligan, R.A., Vale, R.D., and Gibbons, I.R. (2008). Structure and functional role of dynein's microtubule-binding domain. Science *322*, 1691–1695). Their structure and our EM (Figure 2) are consistent, both showing a kink in the coiled coil adjacent to the MTBD.

## Supplemental Data

Document S1. Five Figures and Two Tables

Movie S1. “Scanning” Classification of GFP/BFP-Tagged Constructs (Top Views) Features within a different region around the perimeter of the head were analyzed by using masks with a radial extent of ∼15 nm and excluding features within the head, thus “scanning” the perimeter of the head in search of GFP-based tag positions. The position and shape of the mask are evident from the noise fluctuations visible in the movie. Most positions show only noise, but occasionally globular densities appear corresponding to the inserted GFP and BFP moieties. This “scanning” classification confirms the positions of tags (colored arrowheads) obtained by difference mapping (Figure S2). See full legend in Supplemental Data for further details.

Movie S2. “Scanning” Classification of Right Views See legend to related Movie S1. See full legend in Supplemental Data for further details.

Movie S3. Tilting of the Stalk between Unprimed and Primed Motors Mean stalk positions in primed and unprimed motors are indicated by black and white dashed lines, respectively. The mode of stalk flexibility does not differ between primed and unprimed motors (unlike the stalk of dynein-c), appearing in each case to pivot about the emergence point from the head. See full legend in Supplemental Data for further details.

Movie S4. Movie Showing the 3D Relationship between Tags GN, B1, B2, B5, and B7 tags in the unprimed conformation and the position of GN in the primed conformation are illustrated here for a rotation (*θ*) between top and right views of 90°. The direction of movement of GN between the primed and unprimed conformations relative to top and right views is shown. The magnitude of the 3D movement of GN is between 18.8 and 21.1nm (for *θ* lying between 50° and 116°).
