# Supplementary material for: AAA+ Ring and Linker Swing Mechanism in the Dynein Motor
Source: Cell. 2009 Feb 6;136(3):485–95. doi: 10.1016/j.cell.2008.11.049 (PMC2706395; doi:10.1016/j.cell.2008.11.049)
Supplement: Document S1. Five Figures and Two Tables [file mmc1.pdf]

Cell, Volume *136*

Supplemental Data

AAA+ Ring and Linker Swing Mechanism in the Dynein Motor

Anthony J. Roberts, Naoki Numata, Matt L. Walker, Yusuke S. Kato, Bara Malkova, Takahide Kon, Reiko Ohkura, Fumio Arisaka, Peter J. Knight, Kazuo Sutoh, and Stan A. Burgess

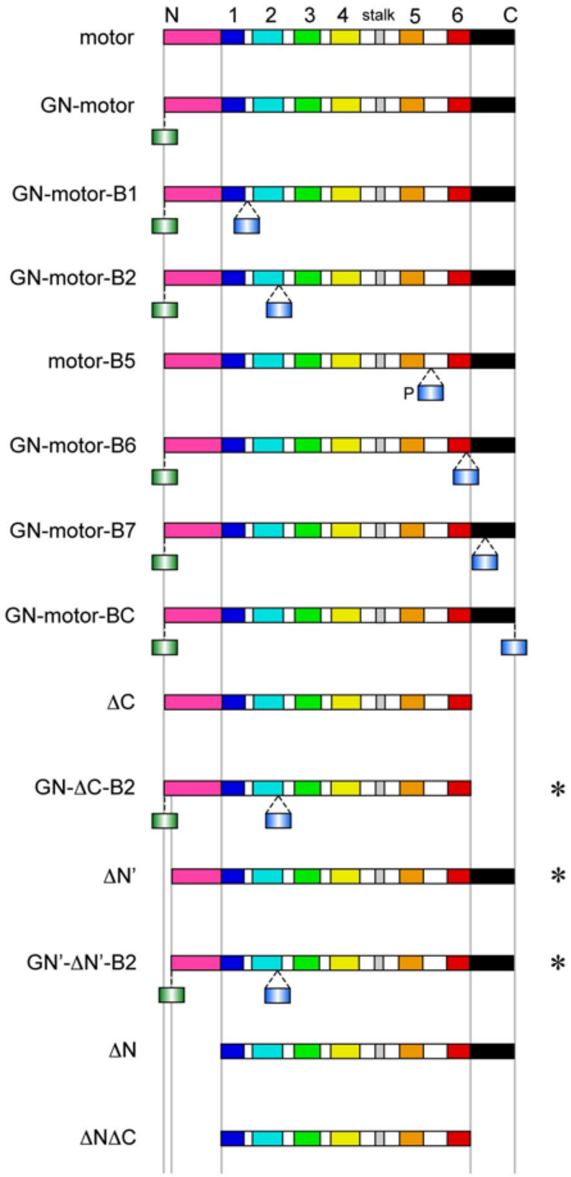

Figure S1 Dynein motor domain constructs used in this study.
Tagged and truncated motor domain constructs are illustrated. GFP and BFP tags are illustrated by green and blue rectangles and their sites of insertion are illustrated with dashed lines. Species shown are: untagged motor domain (residues V1383-I4725, top), motor tagged with GFP at the N-terminus (GN-motor), double-tagged constructs (N-terminal GFP and BFP at various sites within the ring: B1: inserted after A2172; B2: after S2471; B6 after E4261; B7: after S4450 and BC after I4725), single tagged-construct with BFP inserted after a PreScission sequence (P) following K3928 (motor-B5) and truncated constructs (ΔC: V1383-I4319; ΔN': G1459-I4725; ΔN: A1925-I4725 and ΔNΔC: A1925-I4319). Double-tagged truncation constructs (GN-ΔC-B2 and GN'-ΔN'-B2) were used in FRET studies, and along with the ΔN' construct were the only constructs not imaged in this study (*). Color scheme is the same as in Figure 1. Heavy chain sequences are shown to scale. GFP and BFP tags are likely to be flexibly attached to the motor domain given that: 1) the C-terminal residues of GFP/BFP are likely to be disordered in solution (Yang et al., 1996); 2) the sites of insertion have low conservation among dyneins and might correspond to surface loops (Mocz and Gibbons, 2001); and 3) the tags are attached to the motor via four or five flexible spacer residues.

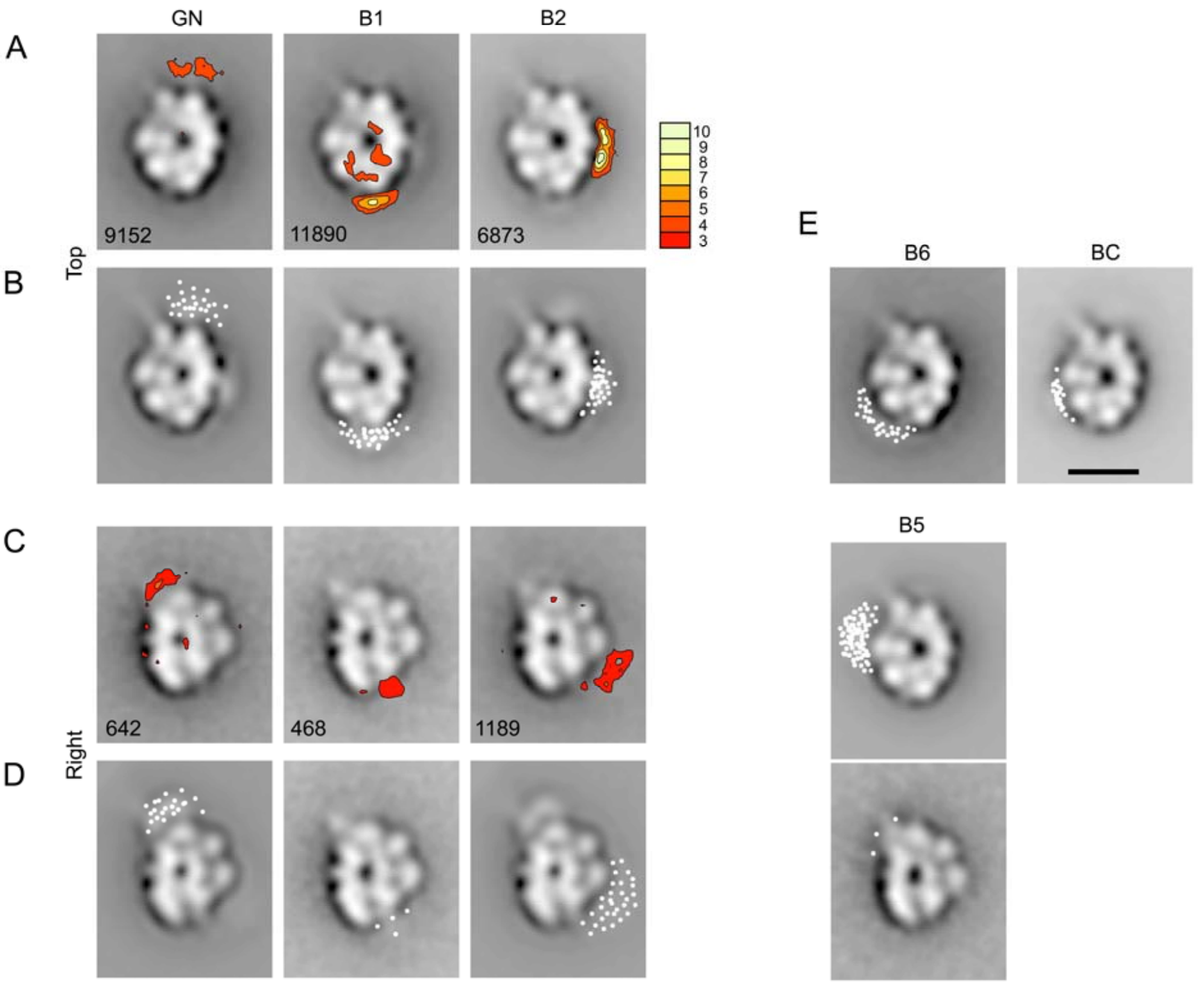

Figure S2 Consistency between tag positions identified by difference mapping and image classification. (**A, C**) Difference mapping to identify tag positions was done by pair-wise subtraction of images as follows: GN: GN-motor minus motor; B1: GN-motor-B1 minus GN-motor; B2: GN-motor-B2 minus GN-motor. For top views (**A**) we subtracted image variances, for right views (**C**) we subtracted image averages, because of the large difference in particle numbers in these two views (numbers in lower left corner). Difference maps are shown contoured at 4σ (**A**) and 3σ (**C**) above the mean and then at intervals of 2σ above these levels (inset: contour levels in σ above the mean). Diffuse, low magnitude differences within the head in B1 top view probably arise from systematic differences in stain quality between GN-motor-B1 and GN-motor and are therefore not significant. (**B, D**) Tag positions were also determined by image classification followed by an automatic detection procedure as follows: from each class average was subtracted the global image average (the latter showing little/very weak features corresponding to tags because of smearing). This revealed the GFP/BFP tag as a bright, roughly circular object whose position was obtained by cross-correlation with a model image of a similarly sized circular object. Those images producing a cross-correlation score above a certain threshold (within a particular view/tag site) had their coordinates recorded and are shown here as scatter plots (white spots). (**E**) Scatter plots showing BFP tag positions in the B5, B6 and BC tagged motors. Scale bar: 10nm.

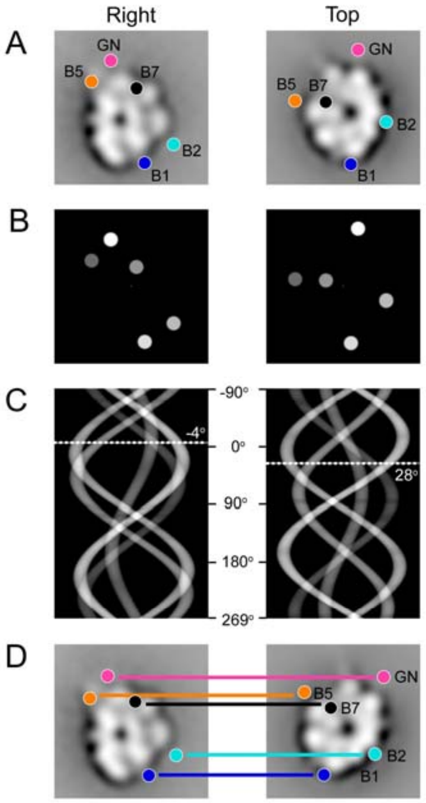

Figure S3 Sinogram analysis used to re-orient right and top views so that they are related to one another by rotation about the y-axis. The principles underlying the procedure are as follows (and see also van Heel et al., (2000)). Any two 2D views of a 3D object can be related to one another by rotation about a single, common axis, but only if each image is correctly oriented on the page so that this axis lies in the same direction in both images. If the two views are each oriented on the page such that this rotation axis lies along the y-axis (i.e. vertically up the page), then it follows that features of the object move only along the x-axis in passing from one view to the other. Discovery of the correct orientation of each image can be achieved by trial and error, but sinogram analysis offers an objective, analytical solution to the problem. Each image is systematically rotated incrementally around the centre of gravity of its features and the image is projected onto the y-axis at each increment to create a 1D projection. There will be a particular angle of rotation of one image for which the 1D projection will have the same profile as the 1D projection of the other image after it has been rotated through a particular, generally different, angle. The movements relating features in the two images at those two rotation angles must then be entirely along the x-axis, and hence the common axis of rotation has been determined and lies along the y-axis in each rotated image.

In our case, the negative staining does not produce a true projection of the dynein head, so features of the head visible as densities in the right view do not necessarily have corresponding densities in the top view. Hence we cannot reliably use features of the head for sinogram analysis. However, the positions of the GFP and BFP tags are unambiguously located in both views, so we have used the mean positions of just these tags to perform the sinogram analysis.

(**A**) Positions of the five tags in each view after shifting to bring the centres of gravity of the tags to the image centre (a requirement for sinogram analysis). (**B**) Images used to generate sinograms have five spots of different brightness, one for each tag. (**C**) For our sinogram analysis we used incremental clockwise rotations of 1° from 0° to 359° to generate a family of 2D images. The IMAGIC sinogram software seeks a horizontal rotation axis rather than the vertical axis we described above. Therefore the family of rotated 2D images are projected onto the x-axis to generate the series of 1D projections, which are then arranged in column to produce a sinogram for each image. Since we wish to define the axis instead as vertical, it is necessary for us to redefine the range 0° to 359°, to be -90° to 269°, as shown. The sinogram correlation function (using the IMAGIC software) is computed (not shown) to find the matching profiles in the two sinograms (dashed lines) and thus the clockwise angle through which each view must be rotated (-4° for the right view and +28° for the top view) in order that the axis of rotation lies along the y-axis. (**D**) The results show that this axis of rotation does indeed account for the simultaneous change in position of all five tags since they all move principally only in the x-direction (colored lines). If distortions had occurred in their positions during specimen preparation some pronounced y-axis displacement could also have been expected, but this is not observed. The small y-axis displacements of B5 and B7 positions are likely caused by small errors in determining their positions: for the small data set of the B5 tag in right view we only found 3 classes, possibly because some were also obscured by the base of the stalk; for the B7 tag we used the peaks of the difference maps. A larger y-axis displacement occurs in the position of the MTBD (not shown) suggesting that the stalk flattens onto the carbon film (probably in top view). This is to be expected given its extended structure. However, significant displacement of tags attached to the head, has not occurred.

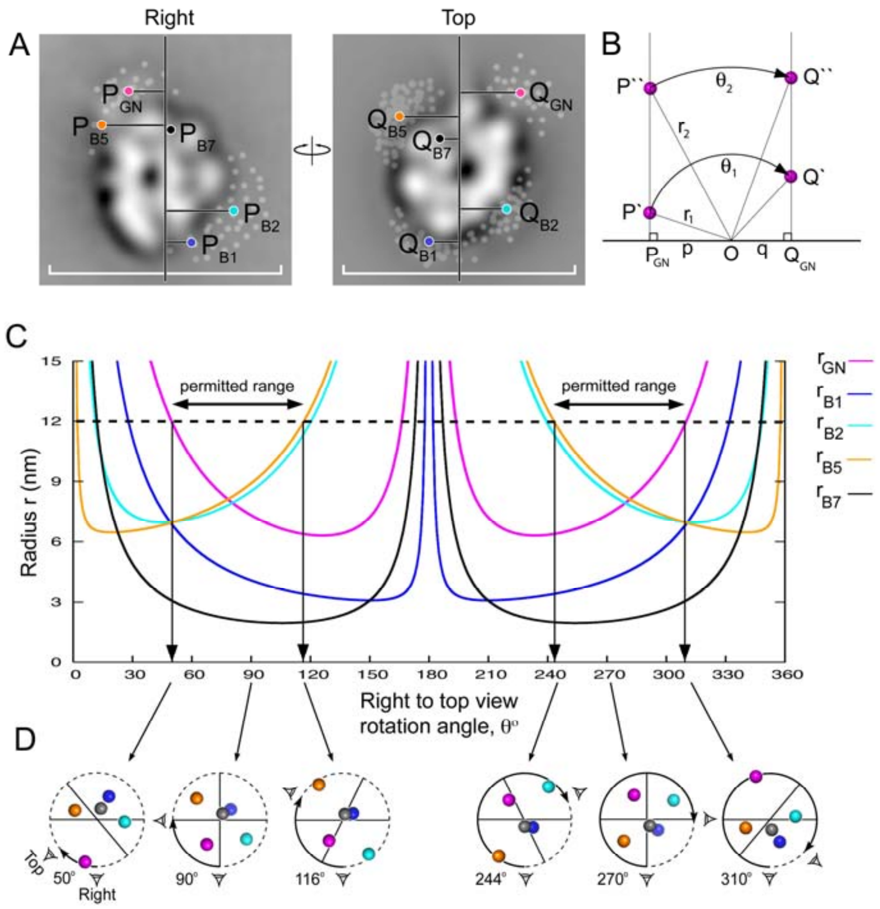

Figure S4 Using tag positions in right and top views to place limits on the angle separating the two views and on the 3D positions of the tags. (**A**) We observe in two views (right & top) the positions of five tags attached to the motor (GN, B1, B2, B5 and B7). We can place limits on the angle relating the two views (θ) by applying the constraint that the tags cannot lie at extreme distances from the rotation axis (vertical lines) in 3D. This is valid because all the tags are attached to the motor which has a diameter of ~13nm. To define the cut-off radius within which the tags must simultaneously lie, we overlaid the positions of all the tags onto each view of the motor (white spots). This led us to choose a radius of 12nm from the rotation axis (white bars), which comfortably contains all the tag positions. (**B**) A schematic diagram looking down the rotation axis (O) showing the relationship between the radius of a tag (e.g. GN as shown here) from the rotation axis (r) and the rotation angle between views (θ). A tag at position P', radius r_1 from the rotation axis rotates through angle θ_1 to position Q', accounting for the projected positions (P and Q) and distances (p, q) from the rotation axis. Other positions with different radii and rotation angles can also account for the projected positions, e.g. a tag at position P'', radius r_2 rotating through angle θ_2 to position Q''. The relationship between radius (r) and rotation angle (θ) is: $r = [(p^2+q^2-2pq\cos\theta)/(\sin^2\theta)]^{1/2}$. (**C**) Plot of r as a function of θ for each of the values of p and q obtained from (**A**). By constraining the values of r for all five markers simultaneously to be $\leq$ 12.0 nm from the rotation axis (dashed line), we obtain two solutions of $50° \leq \theta \leq 116°$ and $244° \leq \theta \leq 310°$ with opposite hands (between which we cannot discriminate, see D). (**D**) Examples of calculated 3D tag positions, viewed down the rotation axis (from the stalk), for different values of θ (indicated). Dashed circle shows 12nm from the rotation axis. Solid lines show planes of top and right views and viewing direction is indicated by the eye symbols. Opposite hands can be seen by comparing 50° to 310°, 90° to 270°, and 116° to 244°. The angle used to illustrate the positions of tags in Figure 6 is 90° because this is easier to illustrate, although the real angle is somewhere between 50°-116° and 244°-310°.

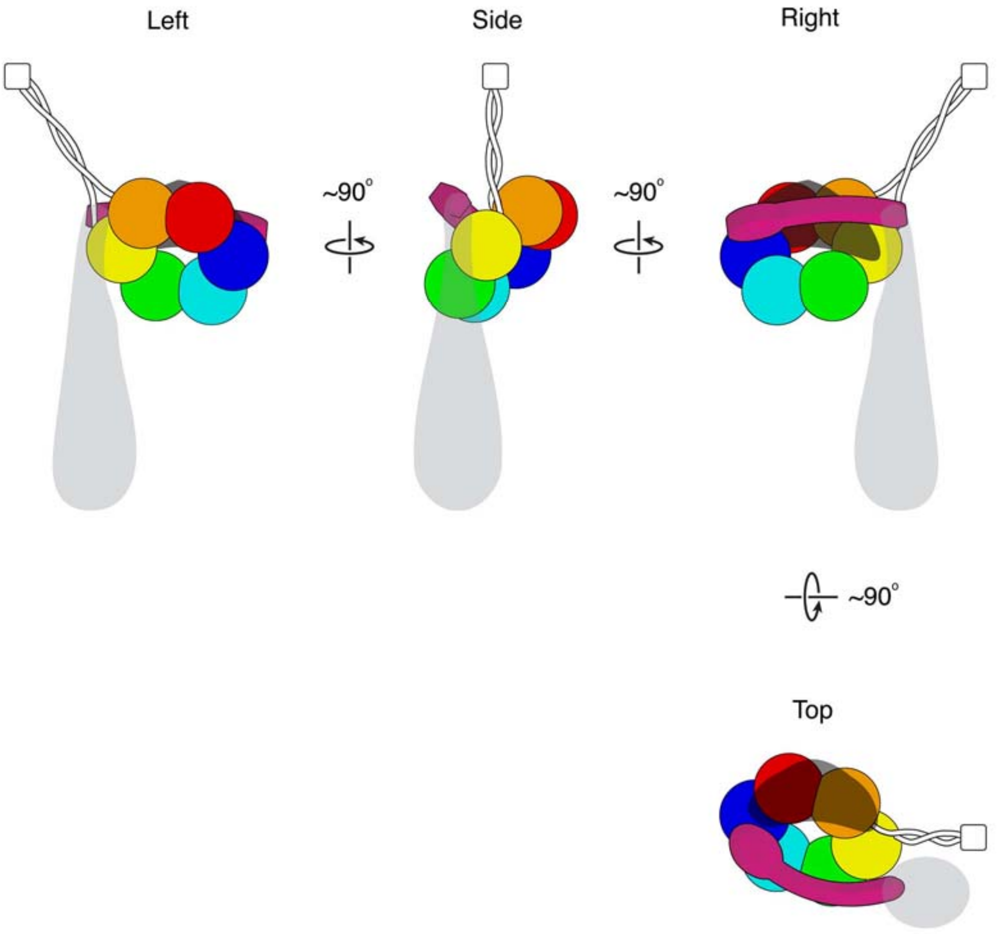

Figure S5 3D interpretation of dynein structure.
The interpretation of top and right views of cytoplasmic dynein in this study is compatible with our earlier interpretation of left, side and right views of dynein-c (Burgess et al., 2004). The full-length tail present in left, side and right views of dynein-c allowed us to interpret these views directly. The tail (grey) is an extension of the linker (magenta). Top and right views described here are related by a rotation about an axis roughly orthogonal to that which relates left, side and right views.

Table S1

Construct	ATPase activity (s^{-1})		MT binding	MT-sliding activity ($\mu m.s^{-1}$)	FRET efficiency (%)		f_m/f_{380}
	Basal	MT-activated			ATP	apo	
motor	5.4 ± 0.3	37.6 ± 1.5	+	3.1 ± 0.3	24.9	7.8	1
ΔC	2.3 ± 0.6	2.5 ± 0.05	-	-	7.5	6.7	0.98
ΔN'	0.6 ± 0.03	2.1 ± 0.07	+	0.06 ± 0.02	14.6	13.7	n.d.
ΔN	< 0.1	< 0.1	-	-	n.a.		0.94
ΔNΔC	< 0.1	< 0.1	-	-	n.a.		0.90

Table S1 Properties of the cytoplasmic dynein truncation constructs.
Microtubule (MT)-activated ATPase and MT binding were determined at 10μM MT. FRET efficiency was measured between BFP on AAA2 and GFP on either the N-terminus of the motor and ΔC, or the truncated N-terminus of ΔN'. Data on the motor is comparable to our previous reports (Kon et al., 2005a; Shima et al., 2006b). f_m/f_{380} is the ratio of frictional coefficients of species *m* and the motor; a value below 1 indicates species *m* is more compact than the motor. The ΔN' construct has an N-terminal truncation of 75 residues (9kDa). The basal ATPase activity of the ΔN' construct is 10-fold slower than the motor, and the impact on microtubule-activated ATPase is more severe. Microtubule binding activity is retained, as is microtubule sliding, albeit at a rate two orders of magnitude lower than the motor. The FRET efficiency from GFP/BFP tagged ΔN' molecules is an intermediate value between the unprimed and primed states of motor, and does not change on the addition of ATP. These results show that the N-terminus of the motor domain is critical for motile activity in *D. discoideum* cytoplasmic dynein. FRET efficiency of GFP/BFP tagged ΔC molecules is similar to the full-length motor in the unprimed state, indicating the absence of a large movement of the N-sequence linker in this construct during steady state ATPase.
n.a. not applicable; **n.d.** not determined.

Table S2

	Unprimed		Primed	
**BFP site**	**Distance from GFP (nm)**	**FRET efficiency (%)**	**Distance from GFP (nm)**	**FRET efficiency (%)**
B1	18.3 – 25.5	3	12.0 – 17.6	20
B2	16.2 – 20.5	5	4.1 – 5.9	36
B5	13.6 – 16.2	6	20.0 – 30.7	4
B7	10.0 – 17.4	13	14.4 – 20.6	5

Table S2 Comparison between FRET efficiencies and 3D distances estimated by EM.
Estimated distances between N-terminal GFP (GN) and four BFPs within the head (B1, B2, B5 and B7). Distances in the primed conformation are calculated on the basis that the positions of B1, B2, B5 and B7 do not change from those in the unprimed conformation. In support of this, we found that the B1 tag position is virtually unchanged (mean displacement of 0.2nm) between primed and unprimed conformations in top view (data not shown). Estimates are from the 3D analysis (Figure 6) shown alongside their corresponding FRET efficiencies from the same constructs (Kon et al., 2005). FRET efficiencies for B5 are reported here for the first time.

Movie S1 ‘Scanning’ classification of GFP/BFP tagged constructs (top views).
For each construct nine independent classifications were performed on all top view images. In each of the nine classifications, features within a different region around the perimeter of the head were analyzed. This was achieved by using nine wedge-shaped masks, each with a radial extent of ~15 nm and an angular extent of 45°, positioned to overlap the preceding one by 5° and excluding features within the head. Each construct was classified into 72 classes. All class averages from each classification were then arranged sequentially to create this movie (9 x 72 frames), thus ‘scanning’ the perimeter of the head in search of GFP-based tag positions. The position and shape of the mask is evident from the noise fluctuations visible in the movie. Most positions show only noise, but occasionally globular densities appear corresponding to the inserted GFP and BFP moieties. This ‘scanning’ classification confirms the positions of tags (colored arrowheads) obtained by difference mapping (Figure S2).

Movie S2 ‘Scanning’ classification of right views (see legend to related Movie S1). Here each construct was classified into 12 classes. Scanning classification shows that in six constructs in top view (Movie S1) and in the four of those constructs seen also in right view (Movie S2), the globular N-terminal GFP (magenta arrowhead) is located near the stalk base in the unprimed conformation. Difference mapping (Figure S2) also yields the same conclusion. Thus two independent objective methods indicate that the N-terminus has a strongly preferred location close to the head, contrary to the earlier report by Meng *et al.* (2006) that it is at random orientations and far from the head. We suggest that in that earlier report, subjective identification of the N-terminal tag in noisy raw images led to an unreliable conclusion. In addition, it is noteworthy that the structure Meng *et al.* identified as the N-terminal ‘tail’ domain in averaged images (their Figure 2) has the same dimensions as the stalk we describe here (Figure 2, Movie S3), suggesting that some of their images may have been rotationally misaligned.

Movie S3 Tilting of the stalk between unprimed and primed motors.
Mean stalk positions in primed and unprimed motors are indicated by black and white dashed lines respectively. The mode of stalk flexibility does not differ between primed and unprimed motors (unlike the stalk of dynein-c), appearing in each case to pivot about the emergence point from the head. Classification of stalks was done in two stages. First we used K-means clustering in SPIDER to produce a series of classes (~50 particles per class) using a mask encompassing all stalk positions. Some classes showed clearly visible stalks. Total number of particles obtained: right view; 3858 (unprimed): 526 (primed); top view: 1604 (unprimed); 1667 (primed). From each of these we obtained manually the coordinates of the centre of the MTBD and the base of the stalk and used these to determine the MTBD’s angle in each class. We then sorted the individual images from all classes into a single list based on their MTBD angle, divided this list into ten partitions and averaged them. Particles per class- right view: 386 (unprimed); 53 (primed); top view: 161 (unprimed); 167 (primed), plus a tenth class containing the remaining images. From these ten image classes we obtained manually the coordinates of the distal stalk (white spots in Figure 5C) and calculated the mean stalk angles (dashed lines).

Movie S4 Movie showing the 3D relationship between tags.
GN, B1, B2, B5 and B7 tags in the unprimed conformation, and the position of GN in the primed conformation are illustrated here for a rotation (θ) between top and right views of 90°. The direction of movement of GN between the primed and unprimed conformations relative to top and right views is shown. The magnitude of the 3D movement of GN is between 18.8 and 21.1nm (for θ lying between 50° and 116°).

Supplemental References

Burgess, S. A., Walker, M. L., Sakakibara, H., Oiwa, K. and Knight, P. J. (2004) The structure of dynein-c by negative stain electron microscopy. J Struct Biol 146, 205-216.

Kon, T., Mogami, T., Ohkura, R., Nishiura, M., and Sutoh, K. (2005). ATP hydrolysis cycle-dependent tail motions in cytoplasmic dynein. Nat Struct Mol Biol 12, 513-519.

Mocz, G., and Gibbons, I. R. (2001). Model for the motor component of dynein heavy chain based on homology to the AAA family of oligomeric ATPases. Structure 9, 93-103.

van Heel, M., Gowen, B., Matadeen, R., Orlova, E. V., Finn, R., Pape, T., Cohen, D., Stark, H., Schmidt, R., Schatz, M., and Patwardhan, A. (2000). Single-particle electron cryo-microscopy: towards atomic resolution. Q Rev Biophys 33, 307-369.

Yang, F., Moss, L. G., and Phillips, G. N., Jr. (1996). The molecular structure of green fluorescent protein. Nat Biotechnol 14, 1246-1251.
