# Supplementary figures and images for: AAA+ Ring and Linker Swing Mechanism in the Dynein Motor
Source: Cell. 2009 Feb 6;136(3):485–95. doi: 10.1016/j.cell.2008.11.049 (PMC2706395; doi:10.1016/j.cell.2008.11.049)

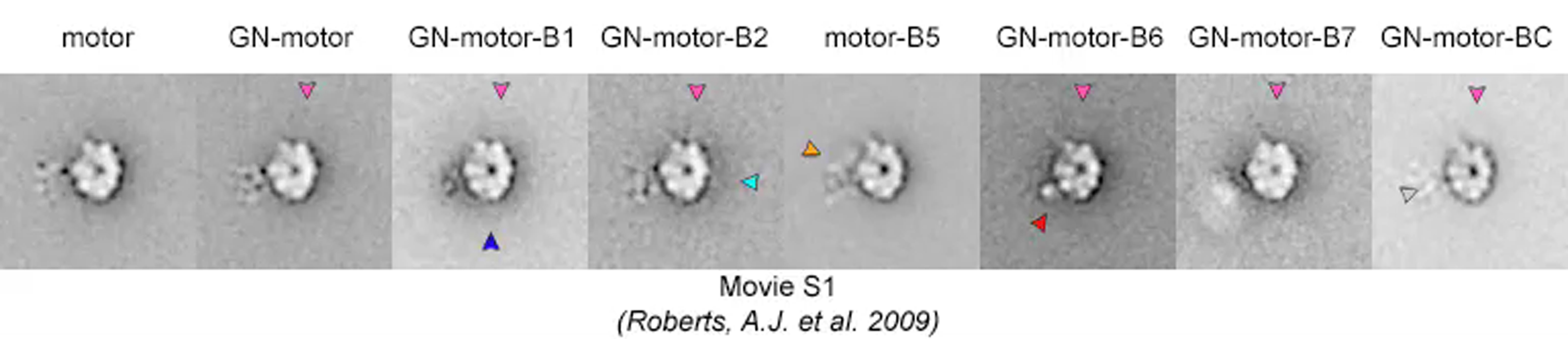

Supplement: Movie S1. “Scanning” Classification of GFP/BFP-Tagged Constructs (Top Views) — Features within a different region around the perimeter of the head were analyzed by using masks with a radial extent of ∼15 nm and excluding features within the head, thus “scanning” the perimeter of the head in search of GFP-based tag positions. The position and shape of the mask are evident from the noise fluctuations visible in the movie. Most positions show only noise, but occasionally globular densities appear corresponding to the inserted GFP and BFP moieties. This “scanning” classification confirms the positions of tags (colored arrowheads) obtained by difference mapping (Figure S2). See full legend in Supplemental Data for further details. [file mmc2.jpg]

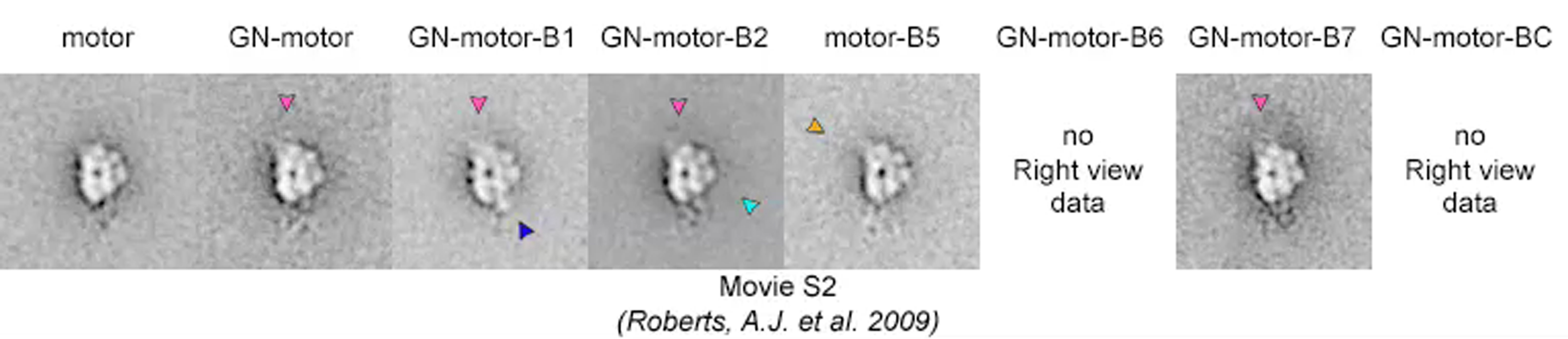

Supplement: Movie S2. “Scanning” Classification of Right Views — See legend to related Movie S1. See full legend in Supplemental Data for further details. [file mmc3.jpg]

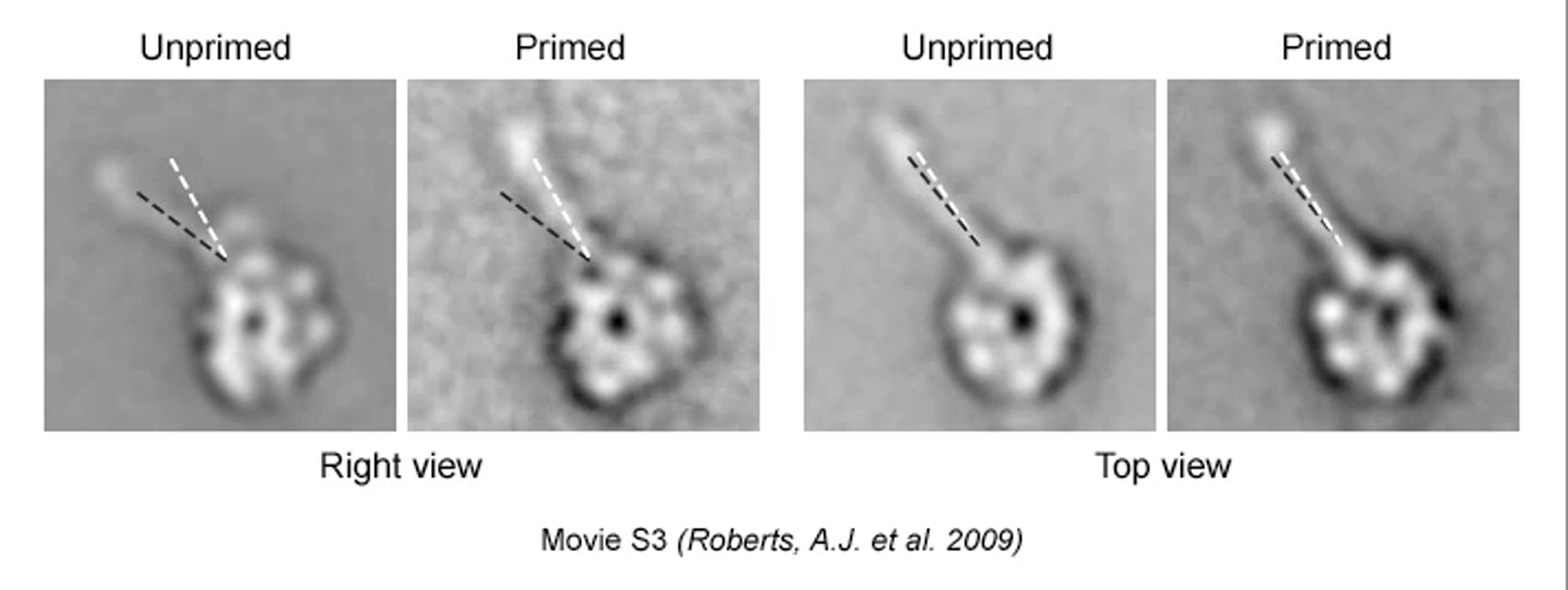

Supplement: Movie S3. Tilting of the Stalk between Unprimed and Primed Motors — Mean stalk positions in primed and unprimed motors are indicated by black and white dashed lines, respectively. The mode of stalk flexibility does not differ between primed and unprimed motors (unlike the stalk of dynein-c), appearing in each case to pivot about the emergence point from the head. See full legend in Supplemental Data for further details. [file mmc4.jpg]

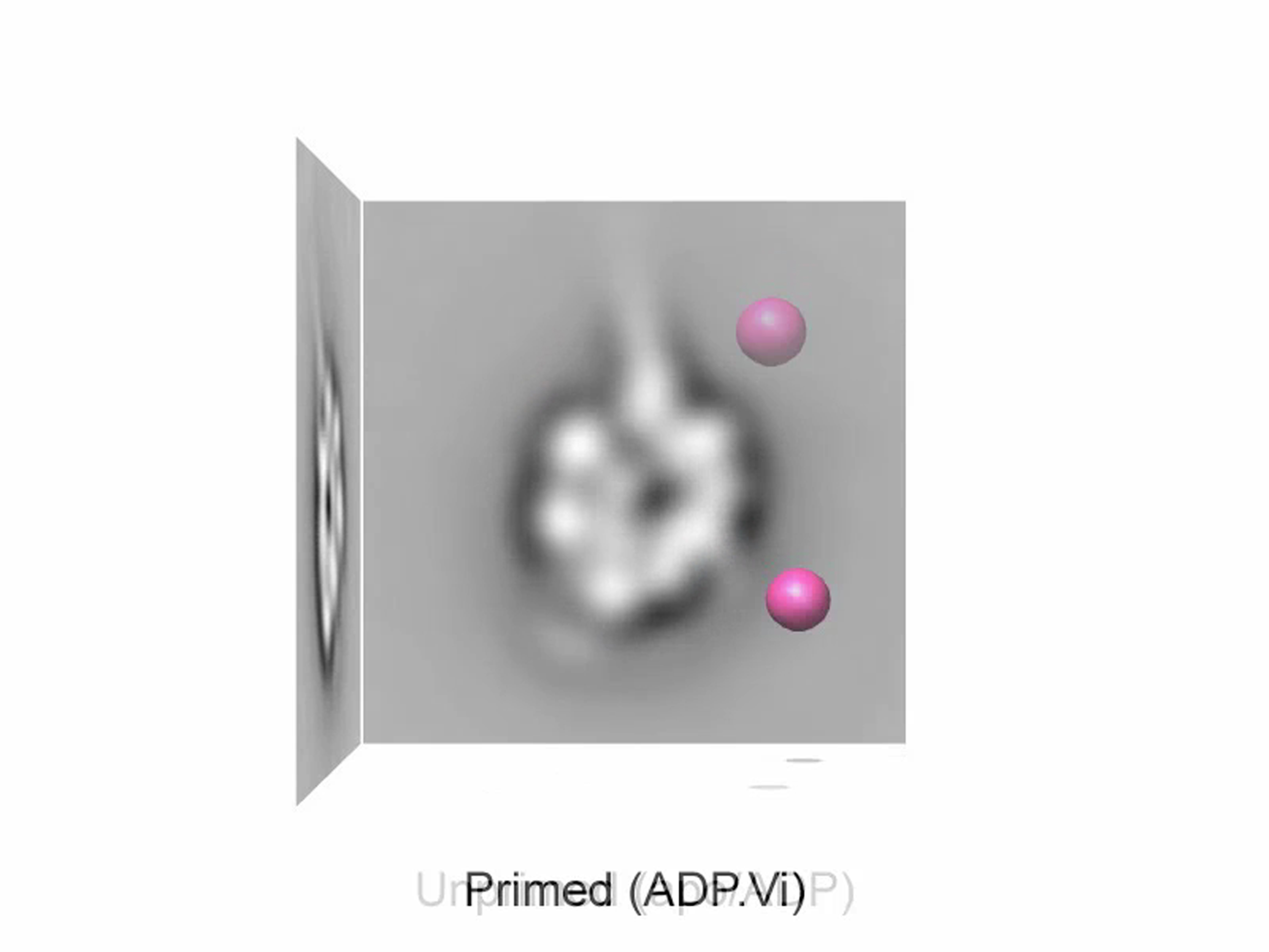

Supplement: Movie S4. Movie Showing the 3D Relationship between Tags — GN, B1, B2, B5, and B7 tags in the unprimed conformation and the position of GN in the primed conformation are illustrated here for a rotation (θ) between top and right views of 90°. The direction of movement of GN between the primed and unprimed conformations relative to top and right views is shown. The magnitude of the 3D movement of GN is between 18.8 and 21.1nm (for θ lying between 50° and 116°). [file mmc5.jpg]
